# Supplementary material for: Unleashing the Biological Potential of Fomes fomentarius via Dry and Wet Milling
Source: Antioxidants (Basel). 2021 Feb 16;10(2):303. doi: 10.3390/antiox10020303 (PMC7920468; doi:10.3390/antiox10020303)
Supplement: Supplementary file 1 [file antioxidants-10-00303-s001.pdf]

antioxidants

MDPI

Supplementary Materials

1. DPPH Assay:

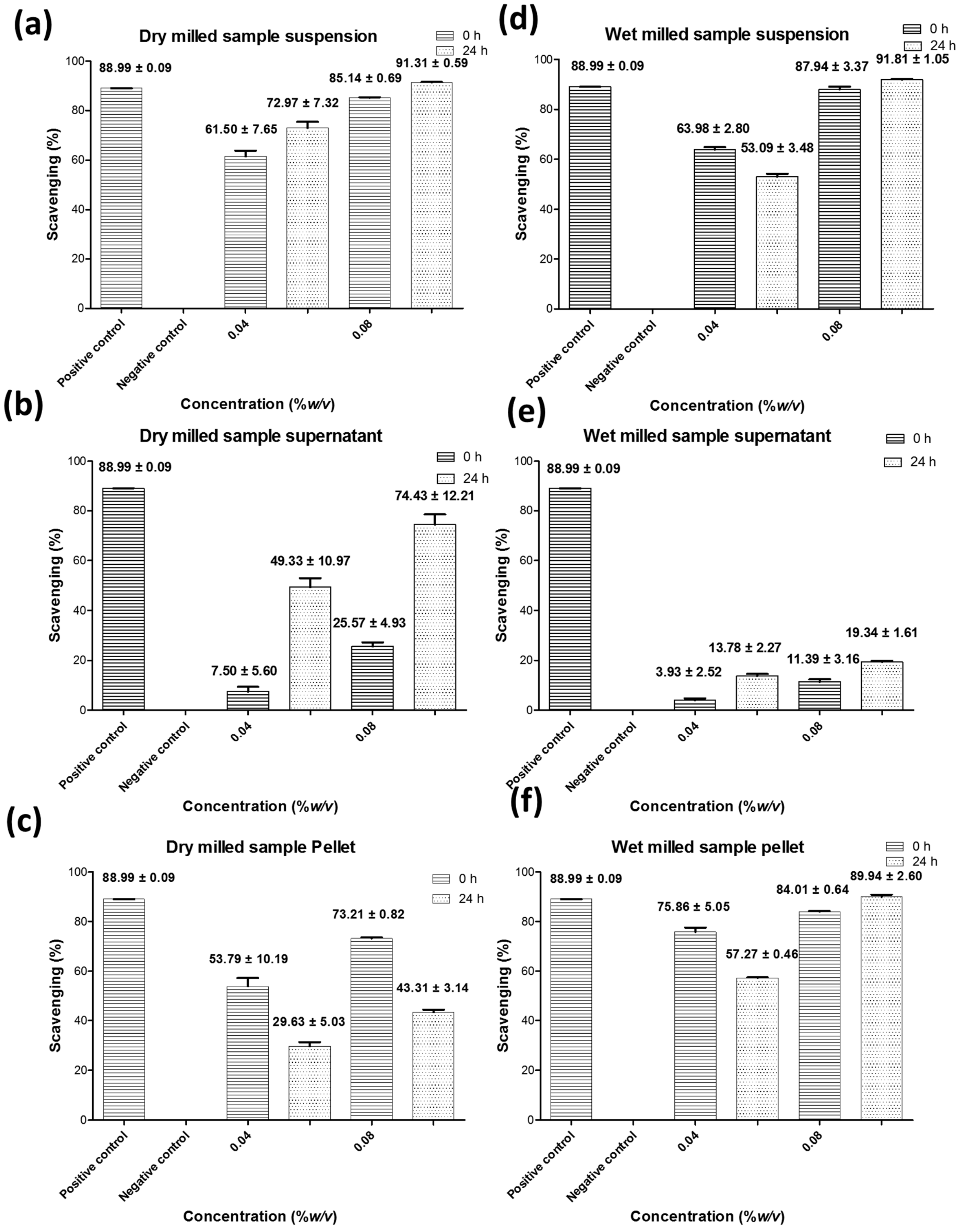

Figure S1. The dry and wet milled samples in suspension scavenge the DPPH radical efficiently even after 24 h of stirring at room temperature. This radical scavenging ability at first is associated primarily albeit not exclusively with the particles them-selves, counting towards a specific surface reactivity of the highly porous microparticles and in general against a

quick diffusion of active ingredients into the surrounding aqueous medium, especially in the case of the wet milled suspension. Dry milled and suspended sample (**a**), supernatant after centrifugation at 19000 g (**b**), resuspended pellet of particles (**c**), wet milled suspension (**d**), supernatant after centrifugation (**e**), resuspended pellet of particles (**f**). Values represent the mean ± SD (n = 3).

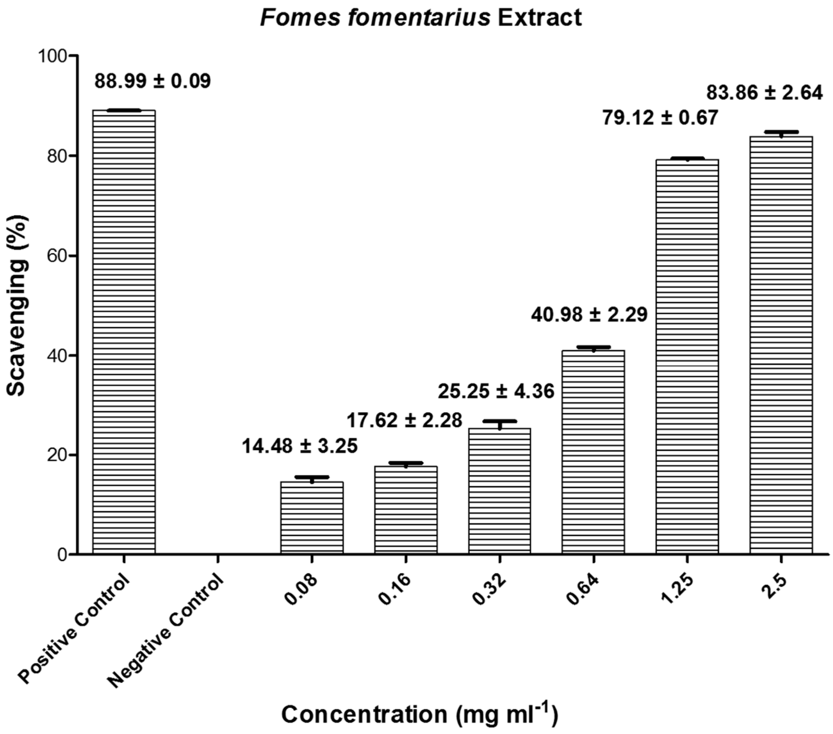

Figure S2. The ethanolic extract *of Fomes fomentarius* scavenges the DPPH radical efficiently in a concentration dependent manner. Values represent the mean ± SD (n = 3).

2. FRAP Assay:

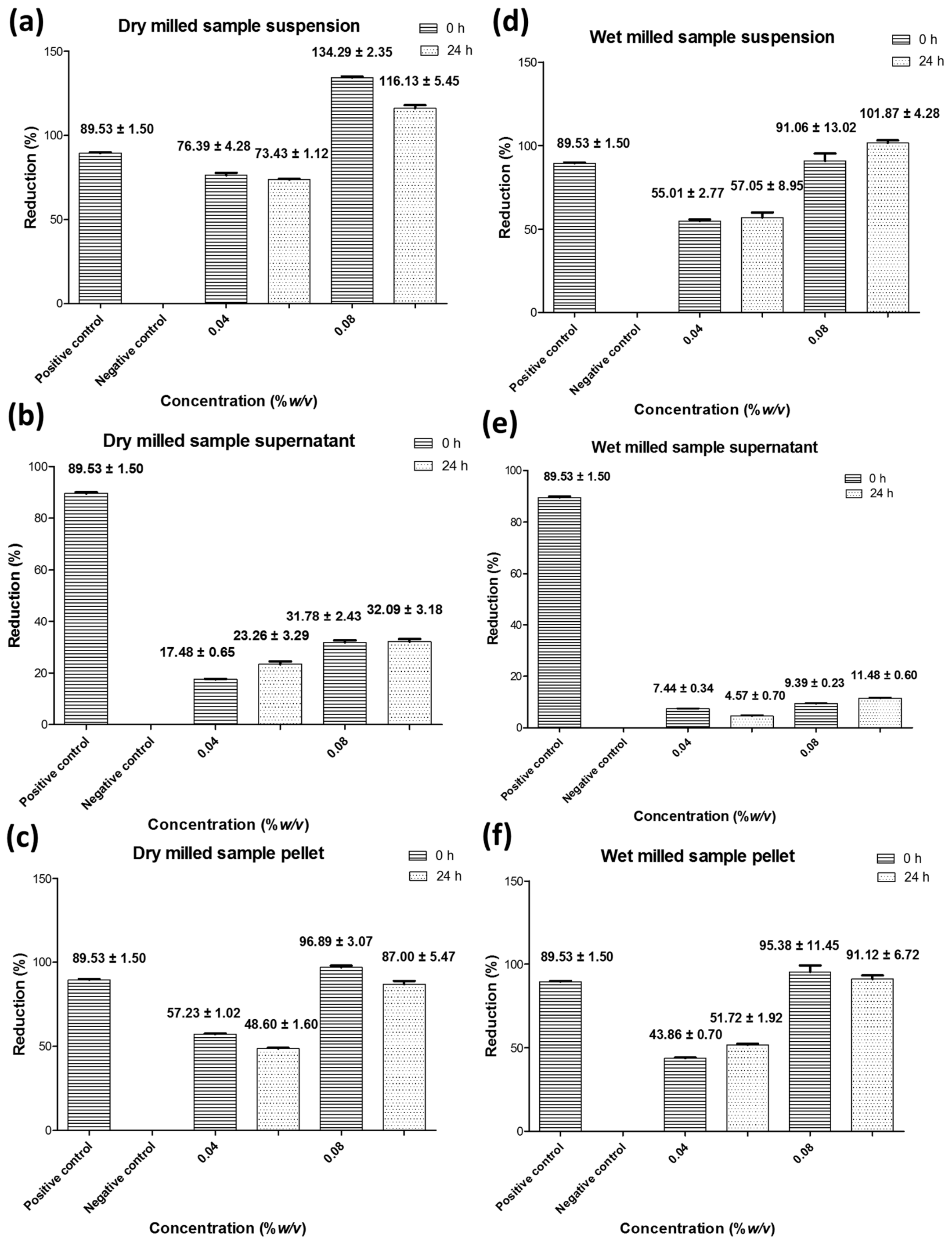

Figure S3. Suspensions of dry and wet milled *Fomes fomentarius* reduce Fe^{3+} ions to Fe^{2+} ions in the ferric reducing antioxidant potential (FRAP) assay even after 24 h of stirring at room temperature. This reducing ability at first is associated primarily albeit not exclusively with the particles themselves, counting towards a specific surface reactivity of the highly porous microparticles and in general against a quick diffusion of active ingredients into the surrounding aqueous medium, especially in the case of the wet milled suspension. Dry milled and suspended sample (**a**), supernatant after centrifugation at 19000 g (**b**), resuspended pellet of particles (**c**), wet milled suspension (**d**), supernatant after centrifugation (**e**), resuspended pellet of particles (**f**). Values represent the mean ± SD (n = 3).

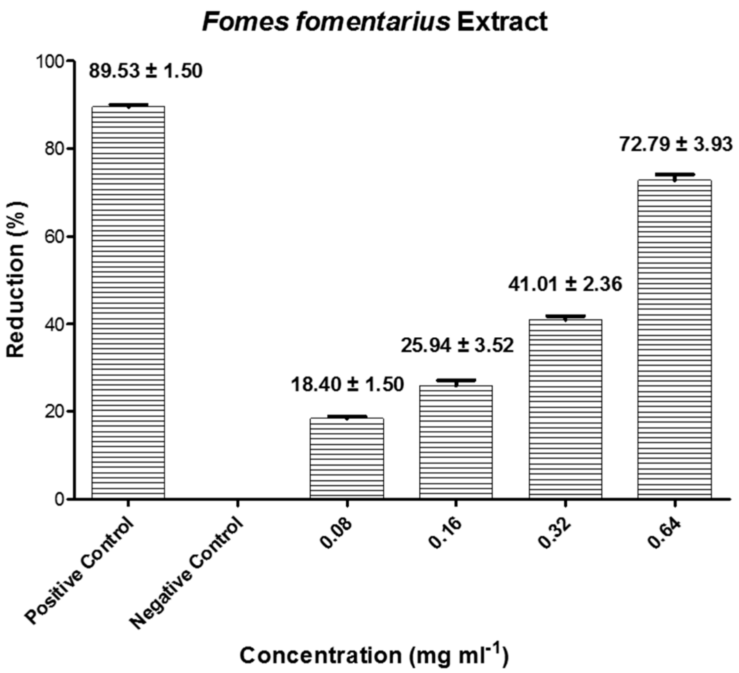

Figure S4. Ethanolic extract of *Fomes fomentarius* reduces Fe^{3+} ions to Fe^{2+} ions in the FRAP assay efficiently in a concentration dependent manner. Values represent the mean ± SD (n = 3).

3. ABTS Assay

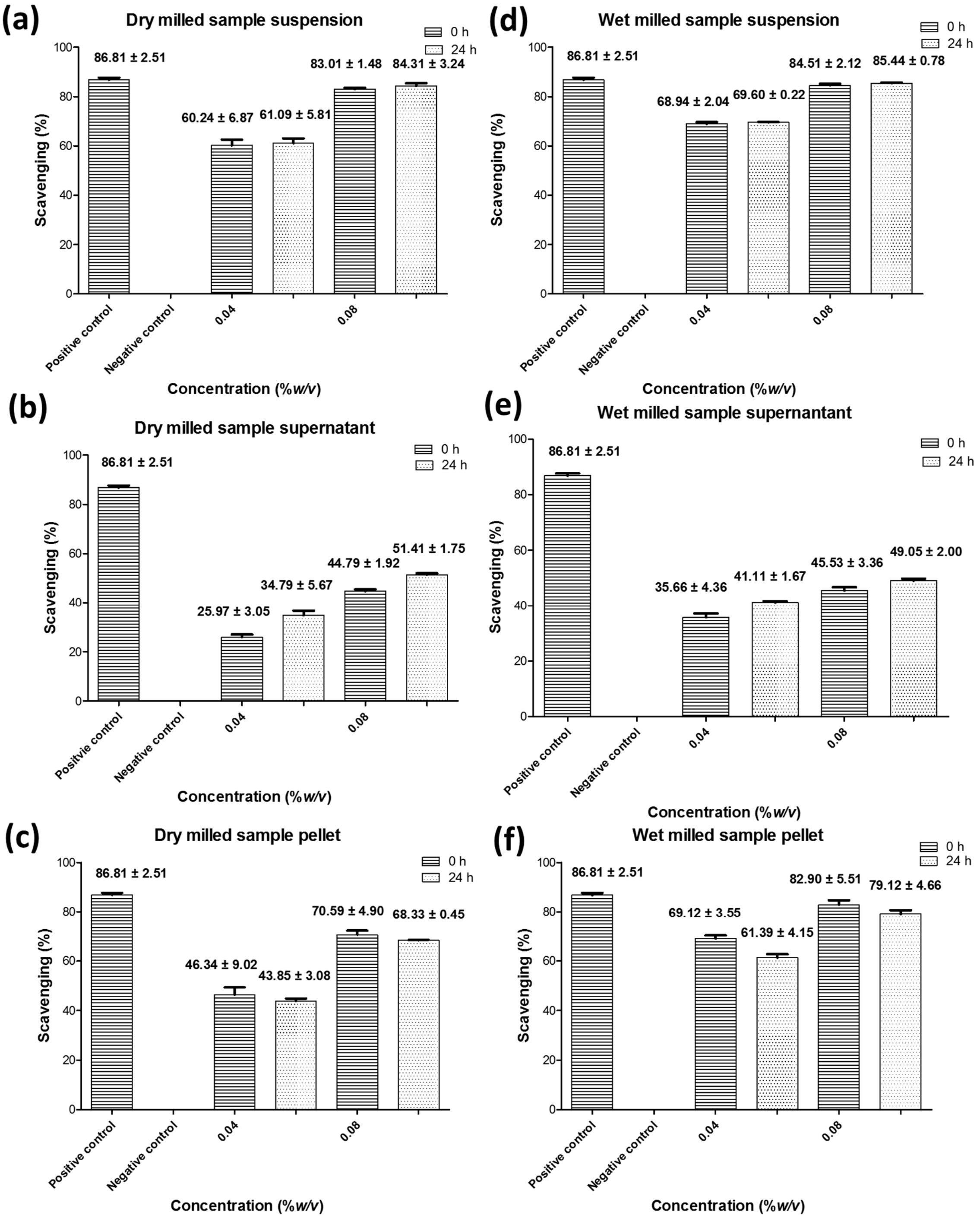

Figure S5. Suspensions of dry and wet milled *Fomes fomentarius* reduce 2,2′-azino-bis(3-ethylbenzothiazoline-6-sulfonic acid ($ABTS^{\bullet +}$) radicals even after 24 h of stirring at room temperature . This reducing ability at first is associated primarily albeit not exclusively with the particles themselves, counting towards a specific surface reactivity of the highly porous microparticles and in general against a quick diffusion of active ingredients into the surrounding aqueous medium, especially in the case of the wet milled suspension. Dry milled and suspended sample (**a**), supernatant after centrifugation at 19000 g (**b**), resuspended pellet of particles (**c**), wet milled suspension (**d**), supernatant after centrifugation (**e**), resuspended pellet of particles (**f**). Values represent the mean ± SD (n = 3).

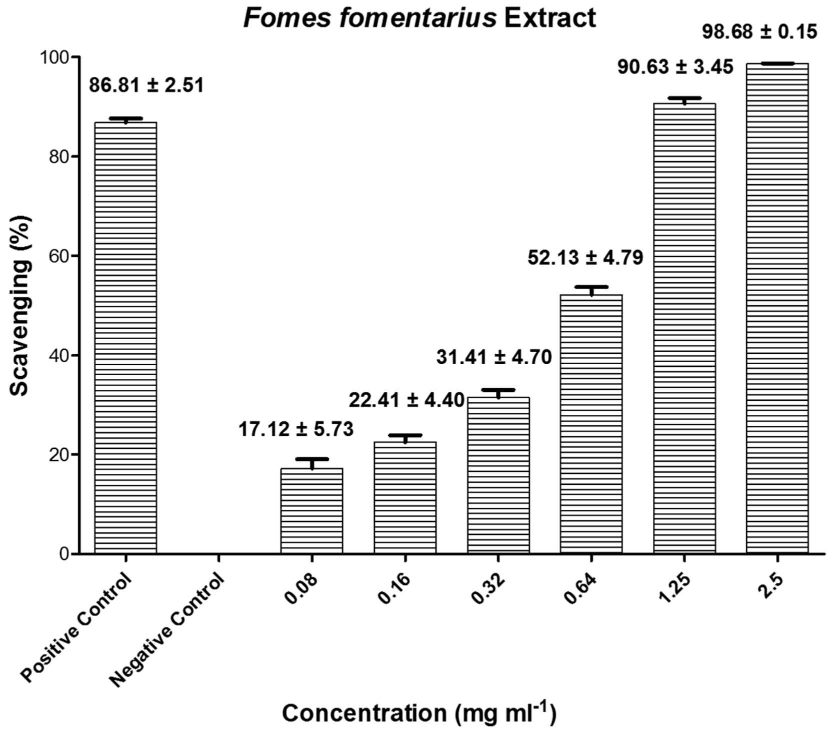

Figure S6. The ethanolic extract of *Fomes fomentarius* scavenges ABTS•+ radicals efficiently in a concentration dependent manner. Values represent the mean ± SD (n = 3).
